# Recent drug approvals for acute myeloid leukemia

**DOI:** 10.1186/s13045-019-0774-x

**Published:** 2019-09-18

**Authors:** Catherine Lai, Kimberley Doucette, Kelly Norsworthy

**Affiliations:** 1 Georgetown University Medical Center, Lombardi Comprehensive Cancer Center, Washington, DC, USA; 2 Center for Drug Evaluation and Research, U.S. Food and Drug Administration, Silver Spring, USA

**Keywords:** Acute myeloid leukemia, Novel treatments, Newly approved drugs

## Abstract

Acute myeloid leukemia (AML) is the most common form of acute leukemia in adults, with an incidence that increases with age, and a generally poor prognosis. The disease is clinically and genetically heterogeneous, and recent advances have improved our understanding of the cytogenetic abnormalities and molecular mutations, aiding in prognostication and risk stratification. Until recently, however, therapeutic options were mostly limited to cytotoxic chemotherapy. Since 2017, there has been an explosion of newly approved treatment options both nationally and internationally, with the majority of new drugs targeting specific gene mutations and/or pivotal cell survival pathways. In this review article, we will discuss these new agents approved for the treatment of AML within the last 2 years, and will outline the mechanistic features and clinical trials that led to their approvals.

## Introduction

As the population across the globe is growing and living longer, more patients are being diagnosed with acute myeloid leukemia (AML) each year. In the United States alone in 2019, there will be an estimated 21,450 new cases of AML diagnosed and 10,920 deaths [1]. With a median age of 68 years and a 5-year overall survival (OS) of roughly 25%, the prognosis remains poor. While 5-year OS is 40% to 50% for younger (< 50 years) patients with de novo AML, the estimated 5-year OS for older patients, those with secondary AML, or relapsed or refractory (R/R) disease is only 5% to 10% [2]. In fact, only about 50% of patients > 60 years receive intensive induction chemotherapy, with the remainder receiving either non-intensive chemotherapy or supportive care [3]. Evaluating trends in epidemiology since 1975, incidence of AML has been slowly increasing, yet the death rate has decreased [1]. The improvement in the death rate over the decades, however, is less linked to improvements in new anti-leukemia drugs then it is to developments in blood banking services, antimicrobials, and management of allogenic hematopoietic stem cell transplant (allo-HSCT) complications [4].

Meanwhile, the advancement of sophisticated molecular technologies over the past 25 years has yielded critical insights into AML pathogenesis and pathophysiology. This molecular characterization continues to expand our understanding of AML biology, mutational patterns that determine the heterogeneity of disease at diagnosis and relapse, and the multiple factors that contribute to lack of response to treatment. Moreover, the descriptive mutational classification has provided a template for development of strategies to target key molecules and pathways in a selective fashion, leading to the development of multiple targeted therapies for the treatment of AML. Perhaps due to the lead-time needed to incorporate our understanding of the molecular underpinnings of the disease, treatment options for AML have been limited for the past five decades. The combination of an anthracycline and cytarabine known as “7 + 3” was initially reported in 1973 [5], and induction therapy has remained relatively unchanged since then. Over the last 40 years, attempts were made to improve “7 + 3” by increasing the dose of anthracycline, alternating the dose and duration of cytarabine, exploring cytarabine given as high-dose short infusions rather than modest-dose continuous infusions, adding mechanistically distinct agents such as etoposide, and giving more or less total chemotherapy doses [6–11]. However, aside from anthracycline approvals in the 1970s to 1990s and tretinoin’s approval for acute promyelocytic leukemia in 1995, no novel agents were approved for AML until 2000, when the FDA granted accelerated approval to gemtuzumab ozogamicin (GO) for older patients with relapsed CD33-positive AML. In 2010, the company voluntarily withdrew GO from the market amidst concerns regarding safety and lack of efficacy on the confirmatory trial [12]. In 2017–2018, the FDA approved a total of eight drugs for AML, including GO at a different dose and schedule. The panoply of new options is exciting for patients and providers alike, but brings with it the challenge of determining optimal sequences and combinations in ways that minimize toxicity and maximize patient benefit.

The purpose of this review is to highlight the recent drug approvals in the United States and internationally in the last 2 years. We will discuss the knowns and unknowns regarding efficacy and safety of these new therapies, including challenges of incorporating them into the current standard of care for diverse molecular and clinical subpopulations and stages of AML.

### FLT3 inhibitors

Fms-related tyrosine kinase 3 (FLT3) mutations are present in about 15–25% of all AML, with a higher percentage in younger patients (≤ 60 years). [2, 13]. There are two defined FLT3 mutations, the FLT3 internal tandem duplication mutation (or ITD subtype) and a FLT3 point mutation in the tyrosine kinase domain (or TKD subtype). FLT3 mutations create proteins that spontaneously dimerize and lead to factor-independent growth, which in mouse models leads to myeloproliferative disorders [14]. About 75% of FLT3 mutations are the ITD subtype, which result in a duplication of between 3 to 100 amino acids located in the juxtamembrane region of the protein. These mutations, especially when there is a high ratio of mutant to wild-type FLT3 alleles and/or ITD insertion in the β1-sheet of the tyrosine kinase 1 domain, are associated with a poor prognosis given high relapse rates and short OS after chemotherapy [2, 15–17]. The remaining 25% of FLT3 mutations are the TKD subtype, which hold an uncertain prognosis [18].

Small molecule inhibitors of FLT3 have achieved mixed results in clinical trials, with first-generation inhibitors studied in R/R AML, showing reductions in blasts but no remissions [19–25]. Since the first FLT-3-directed TKI, CEP-701 (Lestaurtinib) was tested (21), more specific FLT3 inhibitors, such as quizartinib (3% complete remission (CR)) [26] and gilteritinib (discussed below) [27], have led to higher response rates.

### Midostaurin (Rydapt) [28]: newly diagnosed FLT3 mutated AML

In addition to the FLT3-specific small molecule inhibitors, midostaurin has also shown benefit in FLT3-mutated AML. Midostaurin is a multi-targeted kinase inhibitor with activity against FLT3-mutated cell lines in vitro, and in mutant FLT3 xenograft mouse models in vivo [29]. Preclinical development of midostaurin revealed its potential as a protein kinase C (PKC) inhibitor against solid tumors through inhibition of cell proliferation and interruption of cell-cycle activity [30]. Midostaurin and its metabolites generated through the cytochrome p450 pathway target PKC and other serine-threonine and tyrosine kinases [31]. The initial first-in-human trials in R/R FLT3-mutated AML patients found that 70% of patients had a 50% reduction in peripheral blood blasts, but no remissions were observed with a dose of 75 mg three times daily [19]. Subsequently, a phase Ib study evaluating 40 newly diagnosed younger AML patients was done in combination with 7 + 3 using midostaurin at 50 mg twice daily continuously [32]. Gastrointestinal toxicity prohibited use as continuous dosing, but intermittent dosing was found to be tolerable. In this small study, FLT3-mutated patients had similar response rates to those that were FLT3-wild type. The data from this study led to the phase III CALGB RATIFY trial in patients with newly diagnosed FLT3-mutated AML. Patients were randomized 1:1 to receive midostaurin 50 mg twice daily or placebo on days 8–21 in combination with 7 + 3 for up to 2 cycles of induction and in combination with high-dose cytarabine for up to 4 cycles of consolidation, followed by continuous midostaurin or placebo for up to twelve 28-day cycles as maintenance [18]. HSCT could be performed at any time at the discretion of the investigator, at which point treatment with midostaurin was ceased.

While there was only a modest improvement in complete remission on RATIFY (CR; 58.9% midostaurin compared to 53.5% placebo), midostaurin was associated with significantly longer OS (HR 0.78, *p* = 0.009) and event-free survival (EFS; HR 0.78, *p* = 0.002). Benefit was seen for all FLT3-mutated patients, regardless of allelic burden or type of mutation, possibly due to the off-target effects seen with PKC inhibitors [18]. Estimates of median OS were not informative, since the curves for both treatment arms plateaued in the vicinity of 50% after approximately 36 months. The difference in OS was more modest than the medians suggested and was best accounted for by separation of the survival curves around 6 months, when the majority of such patients would be predicted to relapse. One potential explanation for the OS difference is that more patients receiving midostaurin were able to proceed to allo-HSCT in CR1 (28% vs. 23%) (19). It is also possible that the increased durations of OS and EFS with midostaurin could reflect achievement of deeper cell kill resulting in a more durable CR and decreased relapse rates, a hypothesis that is now being tested in follow-up clinical trials [33].

Important grade ≥ 3 adverse reactions or laboratory abnormalities are listed in Table 2. In general, the drug was well-tolerated with only 9% of patients on the midostaurin arm discontinuing secondary to adverse reactions.

In patients eligible for intensive chemotherapy, midostaurin may be added to standard 7 + 3 induction and HiDAC consolidation therapy. However, since the regimen was tested only in newly diagnosed patients < 60 years of age, the role of adding midostaurin to 7 + 3 for newly diagnosed patients age 60 and older is unclear. Given the broad kinase activity of midostaurin, there is an ongoing phase III, randomized, placebo-controlled trial of midostaurin in combination with induction and consolidation chemotherapy in adult patients with newly diagnosed FLT3 mutation-negative AML (NCT03512197). Not only will this trial provide insight into whether a FLT3 mutation is necessary for the efficacy of midostaurin, but given no upper age limit, it should provide insight into the safety of midostaurin in patients age 60 years and older. The use of midostaurin with other cytotoxic chemotherapy agents, or in combination with a hypomethylating agent (HMA), is not approved and needs to be tested in rigorous clinical trials before it can be recommended as a validated approach.

Of note, midostaurin was not granted an indication for maintenance therapy by the FDA, despite inclusion of maintenance therapy on the protocol, yet the EMA included maintenance in the drug’s product information [45]. FDA’s review cited lack of re-randomization prior to maintenance as a major reason that the contribution of maintenance therapy to the treatment effect could not be determined [46]. Results from a post-hoc subset analysis of the RATIFY trial demonstrated no difference in DFS between the treatment arms during the 12 cycles of maintenance (HR = 0.83 [95% CI 0.48–1.43]; *p* = 0.49) and no difference in OS from the time of starting maintenance (HR = 0.96 [95% CI 0.58–1.59]; *p* = 0.86) [47]. Preliminary results of the randomized phase II Radius trial of midostaurin versus standard of care following HSCT in patients with FLT3-ITD-mutated AML (NCT01883362) were recently reported, showing a trend toward increased 18-month relapse-free survival on the midostauin arm [48]. However, confidence intervals were overlapping, and the details of this analysis have not yet been reported. Currently, the data is not sufficiently conclusive to recommend standard of care maintenance therapy with midostaurin following consolidation chemotherapy or HSCT.

### Gilteritinib (Xospata) [41]: relapsed/refractory FLT3 mutated AML

The first-in-human Chrysalis phase I/II (NCT02014558) trial showed that gilteritinib resulted in prolonged responses in FLT3^mut+^ patients with heavily pretreated, refractory, and relapsed AML. Each dose expansion cohort had increasing numbers of FLT3^mut+^ patients [49]. The ADMIRAL trial (NCT02421939) is a recently completed, randomized, open label, multicenter phase III trial of relapsed and refractory FLT3-mutated patients who were randomized 2:1 to receive gilteritinib or salvage chemotherapy (LDAC, azacitidine, MEC, or FLAG-IDA). Randomization was stratified by response to first-line AML therapy and prespecified chemotherapy (intensive vs. low-intensity). FDA approval in 2018 was based on a pre-planned interim efficacy analysis of CR + CRh rate on the gilteritinib arm. A CR + CRh rate of 21% (95% CI of 14.5–28.8) was seen with a median time to response of 3.6 months (range 0.9–9.6 months) and median duration of response of 4.6 months [41]. Transfusion dependence was seen in 77% of patients at baseline and approximately one-third of patients became transfusion independent for at least a 56-day post-baseline period. Of the 23% of patients already transfusion independent prior to the study, more than half (53.1%) remained transfusion independent for at least 56 days post-baseline.

The final OS results from the ADMIRAL trial demonstrated significantly longer median OS of 9.3 months compared to 5.6 months in the salvage chemotherapy arm, and 37.1% compared to 16.7% of patients were alive at 12 months [50], which is encouraging for a single-agent salvage therapy in this high-risk disease subtype. Furthermore, the OS benefit was observed in patients preselected for both high- (HR 0.66 [95% CI 0.47–0.93]) and low-intensity chemotherapy (HR 0.56 [95% CI 0.38–0.84]) [41]. The CR rate was 14.2% versus 10.5% on the gilteritnib versus standard chemotherapy arms, respectively. CR rates were comparable between the arms for patients preselected for high-intensity therapy (15.4% gilteritinib vs. 16% chemotherapy) but were higher on the gilteritinib arm for patients preselected for low-intensity therapy (12% vs. 2%).

Differentiation syndrome (DS) was observed with gilteritinib in 3% of patients, resulting in a boxed warning [41]. DS has been previously described with other FLT3 inhibitors as well and appears to include steroid-responsive neutrophilic dermatoses as a prominent manifestation [51–53]. Other more common and serious adverse reactions are listed in Table 2.

Overall, the results support the use of gilteritinib in patients with R/R AML. The improved OS compared to standard of care chemotherapy options is encouraging. However, response rates remain low. Future research efforts should aim to evaluate combination approaches, particularly for younger patients who can tolerate multi-agent therapy. There are ongoing trials combining gilteritinib with atezolizumab (NCT03730012) and venetoclax (NCT03625505) in patients with R/R AML. Other ongoing studies include randomized comparisons of gilteritinib versus placebo as maintenance therapy post-consolidation (NCT02927262) and post-HSCT (NCT02997202), a randomized comparison of gilteritinib monotherapy versus combination with azacitidine versus azacitidine alone in newly diagnosed AML (NCT02752035), and a trial of gilteritinib in combination with induction and consolidation therapy in patients with newly diagnosed AML (NCT02236013). A randomized phase II trial of gilteritinib versus midostaurin in combination with induction and consolidation chemotherapy is planned (NCT03836209).

### Isocitrate dehydrogenase 1 and 2 inhibitors

Recurrent mutations in IDH1 and IDH2 genes are found in an estimated 7–14% and 8–19% of AML patients, respectively [2]. Mutations in these genes lead to the loss of normal isocitrate dehydrogenase (IDH) catalytic activity and develop neomorphic enzyme activity causing a reduction of α-ketoglutarate to the oncometabolite *R*-2-hydroxyglutarate. This ultimately creates epigenetic alterations and inability of hematopoietic cells to differentiate [54–56]. The prognostic importance of these mutated genes are currently not well elucidated [2].

### Enasidenib (Idhifa) [36]: relapsed/refractory IDH2 mutated AML

A phase I/II clinical trial (NCT01915498) examined the IDH2 inhibitor enasidenib at doses ranging from 50 to 650 mg per day. Based on pharmacokinetic and pharmacodynamic data, 100 mg orally once daily was chosen for the expansion phase. Of 214 patients with R/R AML treated with the 100 mg dose, an overall response (CR + CR with incomplete count recovery [CRi] + CR with incomplete platelet recovery [CRp] + partial remission [PR] + morphologic leukemia free state [MLFS]) was seen in 38.8% of patients (CR 19.6%) with a median response duration of 5.6 months [57]. Time to first response was approximately 2 months and time to CR approximately 4 months. In contrast to more intensive regimens such as 7 + 3, failure to obtain an early response with enasidenib did not necessarily indicate treatment failure. First responses were reported several months after beginning treatment, with median number of cycles received being 5 (range 1–25). Response and survival was similar among patients with IDH2-R140 and IDH2-R172 mutations. The extent of 2-HG suppression correlated with responses in IDH2-R172 patients only, and clearance of mutant IDH2 clones was associated with response [37, 57].

Efficacy for FDA approval was established based on a CR + CR with partial hematologic recovery (CRh) rate of 23% (95% CI of 18–30) and a median remission duration of 8.2 months in 199 adults with R/R AML with the IDH2 mutation per the companion diagnostic test treated with the recommended dose of enasidenib [36]. CRh was defined as less than 5% bone marrow blasts, absolute neutrophil count over 500/μL, and platelet count over 50,000/μL. Furthermore, data on transfusion independence was supportive [58]. Among 157 patients dependent on red blood cell and/or platelet transfusions at baseline, 53 (34%) became independent of transfusions during any 56-day post baseline period.

The most common treatment-emergent adverse event was hyperbilirubinemia (81%, 15% grade ≥ 3), thought to be related to enasidenib’s interference with bilirubin metabolism through inhibition of UGT1A1 [36]. No patients required a dose reduction for hyperbilirubinemia. IDH-inhibitor-associated differentiation syndrome (DS) was reported in 14% of patients as early as 10 days and up to 5 months after enasidenib initiation [36]. However, a recent analysis indicated that the true incidence of any-grade DS was higher at 19% and grade 3 or higher DS was 13%, including two fatalities (1%) [59]. Leukocytosis was seen in 23% of patients [60], with 10% experiencing grade ≥ 3 toxicity, and was observed both independently and in association with 61% of cases of DS [59]. Enasidenib did not appear to cause cytopenias or severe infections.

Overall, enasidenib represents a tolerable treatment option for patients with R/R IDH2-mutated AML. Given the demonstrated transfusion benefit to patients, enasidenib may be particularly useful for older patients unable to tolerate standard cytotoxic agents. It is unknown, however, whether younger patients with R/R IDH2-mutated AML may benefit from a more intensive treatment paradigm. Future trials combining enasidenib with intensive salvage chemotherapy are warranted.

A multicenter phase III clinical trial called IDHENTIFY (NCT02577406) is currently underway comparing the efficacy and safety of enasidenib versus conventional care regimens in subjects 60 years or older with IDH2-positive R/R AML after second or third-line therapy. Other ongoing trials include a phase Ib/2 trial of enasidenib (or ivosidenib) in combination with azacitidine in patients with newly diagnosed IDH-mutated AML (NCT02677922), a phase I trial of enasidenib (or ivosidenib) in combination with induction and consolidation therapy in patients with newly diagnosed IDH-mutated AML, and phase I study of enasidenib maintenance therapy post-HSCT (NCT03515512).

### Ivosidenib (Tibsovo) [61, 62]: newly diagnosed and relapsed/refractory IDH1 mutated AML

A multicenter phase I dose escalation and dose expansion clinical trial with ivosidenib, an oral targeted small molecule inhibitor of mutant IDH1, found clinically significant rates of CR and CRh in patients with R/R AML. Dosing for the expansion cohort was chosen at 500 mg orally daily. At this dose, maximum inhibition of 2-hydroxyglutarate was observed at day 14 in both plasma and bone marrow with no additional inhibition at higher doses [63].

Of 174 adults with IDH1-mutated R/R AML treated with 500 mg ivosidenib daily, the CR + CRh rate was 33% (95% CI 26 to 40) with the rate of CR being 25% [64]. The median duration of CR + CRh was 8.2 months (95% CI 5.6–12.0). Median time to and duration of CR + CRh were 2.0 months and 8.2 months, respectively. A trend toward lower response rates was observed for patients with poor risk cytogenetics, prior HSCT, baseline transfusion independence, two or more prior therapies, and the R132H mutation [62]. The investigators found that clearance of IDH1 mutations (molecular residual disease detected with a sensitivity of 0.02 to 0.04%) was associated with achievement of CR/CRh, and patients who did not respond had enrichment of tyrosine kinase pathway receptor mutations [63]. As with enasidenib, transfusion independence was assessed as a measure of response. Thirty-seven percent of 110 patients who were dependent on red blood cell and/or platelet transfusions at baseline became transfusion independent during any 56-day post-baseline period. Of 64 patients who were independent of both RBC and platelet transfusion at baseline, 59% remained transfusion independent.

Grade 3 or higher adverse reactions in > 5% of patients included DS (13%), QT prolongation (10%), dyspnea (9%), leukocytosis (8%), and tumor lysis syndrome (6%) [36]. Common toxicities are listed in Table 2. Mortality at 30 and 60 days was 7% and 14.3% respectively [63]. Like enasidenib, DS occurred in 19% of patients. Early recognition of DS and treatment with steroids are important to prevent severe and potentially life-threatening complications. If needed, diuretics and hydroxyurea can be used. Leukocytosis occurred in 38% of patients, but only resulted in dose interruption in 3% of patients [61].

Based on these results, ivosidenib is a treatment option for patients with R/R IDH1-mutated AML. Similar to enasidenib, however, it is unknown how the efficacy of ivosidenib compares to other therapies for R/R AML in patients with IDH1 mutations. Further studies are needed to compare efficacy to other standard therapies and to study combinations with other therapeutics in attempt to enhance response rates.

Recently, the FDA expanded the label to include use of ivosidenib for newly diagnosed AML patients age 75 years and older or with comorbidities. The label includes data on a subset of the 34 newly diagnosed patients treated with ivosidenib reported in the original multicenter phase I trial presented by DiNardo et al. [63]. A total of 28 patients with newly diagnosed IDH1-mutated AML age 75 years or older or with comorbidities that precluded the use of intensive induction chemotherapy (e.g., ECOG performance status 2–3, severe cardiac or pulmonary disease, hepatic impairment with bilirubin > 1.5 × upper limit of normal, or creatinine clearance < 45 mL/min) were treated with ivosidenib for a median duration of 4.3 months (range 0.3–40.9) [61]. The CR rate was 28.6% and CR + CRh rate was 42.9%. Median duration of response was not estimable (95% CI 4.2 months—not estimable). Similar to data in the R/R populations, 41.2% of transfusion dependent patients became transfusion independent.

Ivosidenib may be considered for use as an initial single agent for newly diagnosed, elderly AML patients with poor performance status or comorbidities whose leukemia harbors an IDH1 mutation. A potential advantage of this approach is the oral administration. However, DS was more common at 25% in newly diagnosed patients; therefore, adequate precautions must be taken. Furthermore, comparative efficacy data are not available. Recently, phase I data was presented for ivosidenib in combination with azacitidine, showing a CR rate of 57% and CR + CRh rate of 70% [65]. The ongoing multicenter, randomized, phase III clinical trial, AGILE (NCT03173248), will determine the benefit of this approach by comparing azacitidine with or without ivosidenib in adult subjects with previously untreated IDH1-mutated AML not considered candidates for intensive therapy. Still, the question moving forward will be whether ivosidenib + azacitidine is advantageous over venetoclax + azacitidine for first-line therapy of IDH1-mutated AML in patients selected for non-intensive therapy.

### Venetoclax (Venclexta) [44] combinations: newly diagnosed AML ≥ 75 years or comorbidities

B cell lymphoma 2 (BCL-2) is a key regulator of the mitochondrial apoptotic pathway and leads to survival and persistence of AML blasts [66]. BCL-2 sequesters pro-apoptotic BAX, which is released when BCL-2 is antagonized and, in turn, augments permeability of the mitochondrial outer membrane, leading to cell death [66]. Venetoclax is an oral, potent, selective BCL2 inhibitor with proven activity in chronic lymphocytic leukemia (CLL). In AML, BCL2 inhibition is thought to overcome chemotherapy resistance without affecting normal hematopoietic stem cells [67]. The hypomethylating agent (HMA) azacitidine has been shown to reduce levels of MCL-1, an anti-apoptotic protein important in AML cell survival, and a potential resistance pathway for venetoclax [68].

Venetoclax as a single agent in the R/R setting showed little activity with a CR + CRi rate of 19% (CR 6%) and median OS of 4.6 months in the phase II study [69]. However, in patients with IDH1/2 mutations, 33% achieved a CR + CRi. Subsequently, a large multicenter phase Ib dose escalation study (NCT02203773) of venetoclax in combination with HMA (either azacitidine or decitabine) was conducted in treatment-naïve patients age ≥ 65 years who were not eligible for standard induction chemotherapy. Patients with prior HMA therapy or favorable risk cytogenetics were excluded. The overall response (CR + CRi) rate across all venetoclax doses in combination with azacitidine or decitabine was 67%, with a median duration of response of 11.3 months (95% CI 8.9-not reached [NR]), and median OS 17.5 months (95% CI 12.3-NR) [70]. This trial served as the basis for accelerated approval of the combination by FDA in November 2018, with efficacy established based on the rate of CR and duration of CR (see Table 1) in patients age 75 years or older or with comorbidities that precluded the use of intensive induction chemotherapy (defined in the same way as for ivosidenib above) [44]. Notably, the combination was effective in high-risk subgroups: ≥ 75 years, CR + CRi 65% and median duration of response 9.2 months (95% CI 6.4–12.4); adverse genetics (TP53, FLT3-ITD), CR + CRi 60%, median duration of response 6.7 (95% CI 4.1–9.4); and secondary AML, CR + CRi 67% with median duration of response not reached (95% CI 12.5-not reached). Patients with NPM1 and IDH1/2 mutations appeared to have particularly salutary outcomes with this combination (CR + CRi 91% and 71%, respectively) [70]. For patients who obtained CR/CRi and had minimal residual disease (MRD) less than 10^−3^, as measured by multi-parameter flow cytometry, median OS, and duration of response were not reached. When the MRD was greater than 10^−3^, the median OS was again not reached, but median duration of response was 11.3 months. These MRD results require further study to determine their predictive value regarding duration of response and overall outcome.

**Table 1:** Summary of new therapeutic drugs for AML

Newly diagnosed AML								
Drug	Study	Mechanism of action	Indication	Dosing	Country approval	Response Rate (RR) with 95% CI	Median Overall Survival (95% confidence interval)	Duration of Response (DOR) with 95% CI
CPX-351 [34, 35, 71, 72]:	Phase III clinical trial comparing CPX-351 (*n* = 153) to 7 + 3 (*n* = 156)	CPX-351 is a liposomal formulation of fixed 1:5 M ratio encapsulated daunorubicin and cytarabine and it delivers stable synergistic drug ratios to AML cells [73].	t-AML or AML-MRC	Induction:^a^ (daunorubicin 44 mg/m^2^ and cytarabine 100 mg/m^2^) IV, days 1, 3, and 5 Consolidation: (daunorubicin 29 mg/m^2^ and cytarabine 65 mg/m^2^) IV, days 1 and 3	-FDA United States 2017 -EMA 2018	Publication: CR + CRi: 48% vs. 33% in 7 + 3 group CR: 37% versus 26% in 7 + 3 group FDA label: CR: 38% versus 26%	Median OS 9.6 (6.6–11.9) months versus 5.9 (5.0–7.8) in 7 + 3 arm	Not reported
Glasdegib + low dose cytarabine^b^ [42, 74, 75]	BRIGHT AML 1003 phase II randomized clinical trial comparing glasdegib+LDAC (*n* = 77) to LDAC alone (*n* = 38)^c^	Glasdegib is an oral inhibitor of the Hedgehog pathway. It binds to and inhibits Smoothened, a transmembrane protein involved in hedgehog signal transduction. When aberrantly activated, the Hedgehog signaling pathway leads to leukemias by promoting cancer stem cell maintenance. By inhibiting the Hedgehog signaling pathway, leukemic stem cells are reduced [76].	Adults ≥ 75 years or comorbidities that preclude use of intensive induction chemotherapy	Glasdegib 100 mg daily continuously in combination with LDAC^c^	-FDA United States 2018	Publication:^c^ CR 17% versus 2% LDAC arm CR + CRi + MLFS: 27% versus 5% LDAC arm FDA label:^c^ CR 18% (10–29) versus 3% (0.1–14) LDAC arm	Publication:^c^ Median OS 8.8 months versus 4.9 months LDAC arm FDA label: ^c^ Median OS 8.3 months (4.4–12.2) versus 4.3 months (1.9–5.7) LDAC arm	Publication:^c^ CR DOR: glasdegib+LDAC 9.9 months (0.03–28.8)
Midostaurin + standard chemotherapy ^d,e^ [18, 28]	RATIFY phase III clinical trial comparing midostaurin + chemotherapy (*n* = 360) to placebo + chemotherapy (*n* = 357)	Midostaurin is a multi-targeted tyrosine kinase inhibitor that inhibits the activity of wild type FLT3 and FLT3 mutant kinases (ITD and TKD), among others [28].	FLT3 mutation positive as detected by FDA-approved test, in combination with standard cytarabine and daunorubicin induction and cytarabine consolidation	50 mg oral, twice daily with food on days 8 to 21 in combination with Induction: 7 + 3^4^ for up to 2 cycles. Consolidation: HiDAC for up to 4 cycles.^e^ Maintenance: Continuous dosing up to 12 cycles.	-FDA United States 2017 -EMA 2017	Publication: CR 59% (54–64) vs 54% (48–59) in the placebo group	Publication: 74.7 months (31.5-not reached) vs 25.6 months (18.6–42.9) in the placebo arm FDA label: Median survival could not be reliably estimated as survival curves plateaued before reaching the median. HR for OS 0.77 (95% CI 0.63–0.95; *p* = 0.016)	Not reported
Venetoclax + hypomethylating agent^f^ [44, 70]	Study M14–358, non-randomized, phase Ib open-label clinical trial studying venetoclax in combination with azacitidine (*n* = 67) or decitabine (*n* = 13)^g^	Venetoclax is a selective, oral inhibitor of BCL-2 [44] B cell lymphoma 2 (BCL-2) protein plays an important role in the survival and persistence of AML blasts. BCL2 is an important regulator of the mitochondrial apoptotic pathway [66].	Adults ≥ 75 years old or comorbidities that preclude use of intensive induction chemotherapy	Dose ramp-up: 100 mg day 1, 200 mg day 2, 400 mg day 3 and beyond, once daily continuously in combination with azacitidine or decitabine^j^	-FDA United States 2018	Publication:^g^ CR + CRi: 67% CR and CRi rates were 37% and 30% respectively FDA label:^g^ V + aza: CR 37% (26–50), CRh 24% (14–36) V + dec: CR 54% (25–81), CRh 8% (0.2–36)	Publication:^7^ Median OS for all patients was 17.5 months (12.3-NR)	Publication:^g^ CR DOR: 12.5 months (11-NR) CRi DOR: 6.8 months (4.1-NR). CR + CRi DOR: 11.3 months (8.9-NR) FDA label:^g^ V + aza: CR DOR^h^ 5.5 months (range 0.4–30) V + dec: CR DOR^h^ 4.7 months (range 1–18)
Venetoclax + low dose cytarabine^i^ [44, 78, 79]	Study M14–387, phase Ib/II non-randomized, open-label clinical trial studying venetoclax in combination with LDAC (*n* = 61)^j^		Adults ≥ 75 years old or comorbidities that preclude use of intensive induction chemotherapy	Dose ramp-up 100 mg day 1, 200 mg day 2, 400 mg day 3, and 600 mg day 4 and beyond once daily continuously in combination with LDAC^i^	-FDA United States 2018	Publication:^j^ CR and CRi: 54% (42–65) CR: 26% CRi 28% FDA label:^j^ CR: 21% (12–34) CRh: 21% (12–34)	Publication:^j^ Median OS was 10.1 months (5.7–14.2)	Publication:^j^ CR DOR: 8.1 months (5.3–14.9) FDA label:^j^ CR DOR:^h^ 6.0 months (range 0.03–25)
Relapsed/Refractory AML								
Drug	Study	Mechanism of action	Indication	Dosing	Country approval	Response Rate (RR) with 95% CI	Median Overall Survival (95% confidence interval)	Median DOR (95% CI)
Enasidenib [36, 37, 57]	Phase I/II clinical trial studying enasidenib monotherapy (*n* = 199)^k^	Enasidenib is an oral, selective inhibitor of mutant IDH2 enzyme variants R140Q, R172S, and R172K [36]. Mutated IDH2 leads to neomorphic proteins that synthesize 2-hydroxyglutarate, which results in DNA and histone hypermethylation. This in turn blocks cellular differentiation [54–56].	IDH2 mutation as detected by an FDA-approved test	100 mg orally once daily until disease progression or unacceptable toxicity	-FDA United States 2017	Publication: CR: 19.6% (14.5–25.6) CRi/CRp: 9.3% FDA label: CR: 19% (13–25) CRh: 4% (2–8) CR + CRh: 23% (18–30)	Publication: 8.8 months (7.7–9.6)^k^	Publication: DOR: 5.6 months (3.8–7.4) FDA label: CR DOR: 8.2 months (4.7–19.4) CRh DOR: 9.6 months (0.7-NA) CR + CRh DOR: 8.2 (4.3–19.4)
Gilteritinib [41, 80, 81]	ADMIRAL phase III clinical trial comparing gilteritinib (*n* = 247)^l^ to salvage chemotherapy (*n* = 124)	Gilteritinib inhibits multiple receptor tyrosine kinases including FLT3 and demonstrated preclinical activity against FLT3-ITD and FLT3-D835 mutations [41].	FLT3 mutation as detected by an FDA-approved test	120 mg orally once daily	-FDA United States 2018 -PMDA Japan 2018	Interim analysis:^l^ CR or CRh: 21%, (15–29) CR: 12% (7–18) CRh: 9% (5–16) Final analysis: CR 14% (10–19) vs 11% (6–17) in the control group	9.3 (7.7–10.7) months versus 5.6 months (4.7–7.3) for standard chemotherapy	Interim analysis:^l^ CR + CRh DOR: 4.6 months (0.1–15.8) CR DOR: 8.6 months (1–13.8) CRh DOR: 2.9 months (0.1–15.8) Final analysis:^l^ CR DOR: 14.8 months (0.6–23.1+) vs. 1.8 months (< 0.1+ − 1.8) in the control arm
Newly diagnosed and Relapsed/ Refractory (R/R) AML								
Drug	Study	Mechanism of action	Indication	Dosing	Country approval	Response Rate (RR) with 95% CI	Median Overall Survival (95% confidence interval)	Median DOR (95% CI)
Gemtuzumab ozogamicin [38, 82, 83]	Newly diagnosed: Phase III EORTC-GIMEMA AML-19 clinical trial comparing GO (*n* = 118) to best supporting care (*n* = 119)	GO is a CD-33 directed ADC. The drug conjugate consists of a small molecule portion, N-acetyl gamma calicheamicin, which is a cytotoxic agent covalently bound to the antibody via a linker. GO acts through binding of the ADC to CD33-expressing cancer cells [38].	Newly diagnosed: CD33-positive AML in adults	Newly diagnosed: Induction: 6 mg/m^2^ IV on day 1 and 3 mg/m^2^ IV on day 8 Continuation: 2 mg/m^2^ IV day 1 every 4 weeks for up to 8 cycles	FDA United States 2017	Newly diagnosed: CR: 15% CRi: 12% CR + CRi: 27%	Newly diagnosed: 4.9 months (4.2–6.8) versus 3.6 months (2.6 to 4.2 months) in the best supportive care group	Newly diagnosed: Median DFS CR/CRi patients 5.3 months (95% CI 3.1–8.0)
	R/R: MyloFrance-1, phase II, single-arm, open-label clinical trial studying GO monotherapy (*n* = 57)		R/R: CD33 positive AML in adults and pediatric patients 2 years and older	R/R: 3 mg/m^2^ (up to one 4.5 mg vial) IV on days 1, 4, and 7 of a single course		R/R: CR 26% (16–40%) CR and CRp 33%	R/R: Median OS 8.4 months	R/R: Median RFS CR patients: 11.6 months
Gemtuzumab ozogamicin (GO) + standard chemotherapy [12, 38, 77, 84]	Newly diagnosed: ALFA-0701: a randomized, open-label, phase III study comparing GO+ 7 + 3 (*n* = 135) to 7 + 3 alone (*n* = 136)^m^		Newly diagnosed: CD33-positive AML in adults	Newly diagnosed: Induction: 3 mg/m^2^ (up to one 4.5 mg vial) IV days 1, 4, and 7 in combination with 7 + 3^n^ Consolidation: 3 mg/m^2^ (up to one 4.5 mg vial) IV day 1 in combination with daunorubicin and cytarabine for up to 2 cycles^o^	-FDA United States 2017 -European medicines agency 2018	Newly diagnosed: CR: 73% versus 72% in 7 + 3 group. CRp: 8% versus 3% in 7 + 3 group CR and CRp: 81% versus 75% in the 7 + 3 group	Newly diagnosed: 25.4 months versus 20.8 months in the 7 + 3 group^p^	Newly diagnosed: Not reported
Ivosidenib [61, 63]	Phase I/II clinical trial studying ivosidenib monotherapy (*n* = 174 R/R, *n* = 28 newly diagnosed)^q^	Ivosidenib is a small molecule inhibitor that targets the mutant IDH1 enzyme. This targeted treatment is a potent inhibitor of 2-hydroxyglutarate, a neomorphic protein produced by a mutated IDH1 enzyme. 2-hydroxyglutarate competitively inhibits α-ketoglutarate–dependent enzymes and causes epigenetic alterations and impaired hematopoietic differentiation [54–56]. Ivosidenib leads to differentiation and maturation of malignant cells [54–56].	Newly diagnosed: Adults ≥ 75 years or with comorbidities that preclude use of intensive induction chemotherapy	500 mg orally daily until disease progression or unacceptable toxicity	Newly diagnosed: FDA United States 2019	Newly diagnosed: Publication:^q^ CR: 21% (9–38) CR + CRh: 35% (20–54) FDA label:^q^ CR: 29% (13, 49) CR + CRh: 43% (25, 63).	Newly diagnosed: Not reported	Newly diagnosed: Publication:^q^ CR DOR: NE (5.6-NE) CR + CRh DOR: NE (1.0-NE) FDA label:^q^ CR DOR: NE (4.2-NE) CR + CRh DOR: NE (4.2-NE)
			R/R: IDH1 mutation as detected by an FDA-approved test		R/R: FDA United States 2018	R/R: Publication:^q^ CR: 22% (16–29) CR or CRh: 30% (24–38) FDA label:^q^ CR: 25% (19–32) CRh: 8% (5–13) CR + CRh: 33% (26–40)	R/R: Publication: 8.8 months (6.7 to 10.2)^r^	R/R: Publication:^q^ CR DOR: 9.3 months (5.6–12.5) CR + CRh DOR: 6.5 months (5.5–11.1) FDA label:^q^ CR DOR: 10.1 months (6.5–22.2) CRh DOR: 3.6 months (1–5.5) CR + CRh DOR: 8.2 months (5.6–12)

*7 + 3* 7 days continuous infusion cytarabine in combination with 3 days intravenous daunorubicin, *ADC* antibody-drug conjugate, *AML* acute myeloid leukemia, *AML*-*MRC* AML with myelodysplasia-related changes, *aza* azacitidine, *CI* confidence interval, *CR* complete remission, *CRh* complete remission with partial hematologic recovery, *CRi* complete remission with incomplete count recovery, *dec* decitabine, *EMA* European Medicines Agency, *FLT3* Fms-like tyrosine kinase 3, *FDA* Food and Drug Administration, *HiDAC* high dose cytarabine, *ITD*, internal tandem duplication *IV* intravenous; *LDAC* low-dose cytarabine, *MLFS* morphologic leukemia free state, *NA* not available, *NE* not estimable, *NR* not reached, *OS* overall survival, *RFS* relapse-free survival, *t*-*AML* therapy-related AML; *TKD* tyrosine kinase domain, *V* venetoclax

^a^Note that the second induction (for patients failing for achieve a response with the first induction cycle) uses the same dose of (daunorubicin 44 mg/m^2^ and cytarabine 100 mg/m^2^), but on days 1 and 3 only

^b^Cytarabine 20 mg subcutaneously twice daily days 1–10 of each 28-day cycle

^c^Results in [75] presented data on 132 total patients randomized to glasdegib + LDAC (*n* = 88) or LDAC (*n* = 44), including patients with high-risk MDS, a condition for which glasdegib is not approved. The FDA label included the *N* = 115 patients with confirmed AML randomized to glasdegib + LDAC (*n* = 77) or LDAC (*n* = 38) [85]

^d^Daunorubicin dosed at 60 mg/m^2^ IV daily on days 1–3. Cytarabine dosed at 200 mg/m^2^ continuous infusion days 1–7

^e^HiDAC dose was 3 g/m^2^ IV every 12 h on days 1, 3, and 5

^f^Azacitidine 75 mg/m^2^ IV or subcutaneous days 1–7 of each 28-day cycle. Decitabine 20 mg/m^2^ IV days 1–5 of each 28-day cycle

^g^Results in [70] included 145 total patients treated with azacitidine or decitabine in combination with different dose levels of venetoclax. The FDA label included *N* = 80 patients who were age 75 or older or who had comorbidities that precluded the use of intensive induction chemotherapy and were treated with the recommended dose of venetoclax in combination with azacitidine (*n* = 67) or decitabine (*n* = 13) [44]

^h^DOR defined as the median observed time in remission, i.e. the time from the start of CR to the time of data cut-off date or relapse from CR. Median follow-up was 7.9 months (range 0.4–36) for azacitidine, 11 months (range 0.7–21) for decitabine, and 6.5 months (range 0.3–34) for LDAC [44]

^i^Cytarabine 20 mg/m^2^ subcutaneously once daily days 1–10 of each 28-day cycle

^j^Results in included all 82 patients treated with the recommended dose of venetoclax in combination with LDAC. The FDA label included *n* = 61 patients who were age 75 or older or who had comorbidities that precluded the use of intensive induction chemotherapy [44]

^k^Results in had cut-off of September 1, 2017 and presented data on all 214 patients treated with enasidenib at the recommended dose. The FDA label included *N* = 199 patients with IDH2 mutation per the companion diagnostic test treated with the recommended dose, with a data cut date of October 14, 2016 [58]

^l^The FDA label includes an interim analysis of CR + CRh in *N* = 138 patients randomized to the gilteritinib arm that were at least four treatment cycles past the first dose of gilteritinib at the time of the first pre-specified interim analysis [80]

^m^Results in [77, 84] included 278 randomized patients. FDA considered *n* = 271 patients in a modified intent-to-treat population based on lack of documentation of informed consent in the remaining patients [12]

^n^Daunorubicin dosed at 60 mg/m^2^ IV days 1–3. Cytarabine dosed at 200 mg/m^2^ continuous infusion days 1–7. Patients could receive a second induction with daunorubicin and cytarabine alone (i.e., no GO for second induction course)

^o^Daunorubicin dosed at 60 mg/m^2^ IV day 1 of consolidation course 1 and days 1–2 of consolidation course 2. Cytarabine dosed at 1 g/m^2^ every 12 h on days 1–4

^p^Note that the final analysis did not demonstrate a statistically significant benefit in median OS. FDA approval was on the basis of an EFS benefit of 13.6 months GO arm vs. 8.8 months 7 + 3 arm [12]

^q^Results in [63] had cut-off of May 12, 2017 and presented data on 179 patients with R/R AML and 34 patients with newly diagnosed AML treated with ivosidenib at the recommended dose. The FDA label included *N* = 174 patients with R/R AML and *N* = 28 patients with newly-diagnosed AML (the latter with age ≥ 75 years or comorbidities that precluded use of intensive induction chemotherapy) with confirmed IDH1 mutation per the companion diagnostic test and used a data cut-off date of November 10, 2017 in R/R patients [86] and a later data cut-off for newly-diagnosed patients (not yet public, but FDA review will be available soon at https://www.accessdata.fda.gov/scripts/cder/daf/)

^r^OS reported for the primary efficacy population, which included the first 125 patients with R/R AML with second or later relapse, relapse after stem-cell transplantation, disease refractory to induction or reinduction chemotherapy, or relapse < 12 months after initial therapy who received the recommended dose and whose first dose of ivosidenib was at least 6 months before the analysis cut-off date

There was a higher frequency of adverse effects at the 800 mg and 1200 mg doses, and 400 mg was the chosen dose for the phase III trial and FDA approval. Even at the 400 mg dose, recurrent grade 3 and 4 neutropenia required management with dose interruptions, reduction in dosing duration, and/or delays in treatment cycles. The most common (> 10%) grade ≥ 3 adverse reactions and laboratory abnormalities in patients treated with venetoclax in combination with HMAs are listed in Table 2. Unlike CLL patients, tumor lysis syndrome (TLS) was not observed on the trial, but all patients received ramp-up dosing of venetoclax during cycle 1, were hospitalized for at least 3 to 5 days, and received TLS prophylaxis for at least 72 h prior to dosing.

**Table 2:** Toxicities of new therapeutic drugs for AML

Drug	Toxicity	Timing	Treatment
CPX-351 [34]	Common (≥ 25% incidence and ≥ 2% more common on CPX-351 arm): Hemorrhage (70% vs. 49%), rash (54% vs. 36%), constipation (40% vs. 38%), musculoskeletal pain (38% vs. 34%), abdominal pain (33% vs. 30%), cough (33% vs. 23%), headache (33% vs. 24%), arrhythmia (30% vs. 27%), and pneumonia (26% vs. 23%). Prolonged thrombocytopenia (28% CPX-351 vs. 12% 7 + 3), prolonged neutropenia (17% vs. 3%)^a^	During induction phase Platelet recovery^b^ 35 vs. 29 days Neutrophil recovery^b^ 36.5 vs. 29 days [35]	Supportive care: Monitor blood counts frequently until recovery. Administer platelet transfusions as needed. Treat with anti-microbials per institutional standards
	Serious: 1) Hemorrhage (> grade 3 12% vs 8%) 2) Cardiotoxicity 3) Hypersensitivity reactions 4) Copper overload	During the entire treatment period.	1) Supportive care: Monitor blood counts frequently until recovery. Administer transfusions as needed. 2) Check Echo at baseline and before consolidation. 3) Interrupt infusion immediately for hypersensitivity reactions. For mild symptoms, reinitiate the infusion at half the prior rate and consider premedication with antihistamines and/or steroids for subsequent doses. For moderate symptoms, do not reinitiate and premedicate prior to subsequent doses. For severe/life-threatening symptoms, permanently discontinue. 4) Caution in treating patients with Wilson’s disease or other copper-related metabolic disorders.
Enasidenib [36]	Common adverse reactions and laboratory abnormalities (≥ 30% all-grade; ≥ 5% grade ≥ 3): total bilirubin increased (81%; 15%), hypocalcemia (74%; 8%), nausea (50%; 5%), diarrhea (43%; 8%), hypokalemia (41%; 15%), vomiting (34%; 2%), decreased appetite (34%; 4%), tumor lysis syndrome (6%; 6%), differentiation syndrome (14%; 7%); non-infectious leukocytosis (12%; 6%).	During the entire treatment period.	If bilirubin > 3 times upper limit of normal (ULN) for ≥ 2 weeks with no other suspected etiology or elevation in transaminases, reduce dose to 50 mg daily. Resume at 100 mg daily if bilirubin resolves to less than 2 × ULN.
	Serious: 1) Differentiation syndrome, 2) Non-infectious leukocytosis	1) Differentiation syndrome was seen from 10 days to 5 months after starting therapy 2) Non-infectious leukocytosis is typically seen in the first 2 cycles of treatment [37]	1) Steroids (Dexamethasone 10 mg BID) with taper and supportive care. Interrupt drug if intubation or ventilator support are required and/or kidney dysfunction persists > 48 h. Resume when adverse events are ≤ grade 2. 2) Initiate treatment with hydroxyurea and interrupt drug if leukocytosis does not improve. When WBC < 30 × 10^9^/L, resume drug.
Gemtuzumab ozogamicin [38–40]	Common (≥20%) monotherapy^c^: fever (79%), infection (42%), increased AST (40%), bleeding (23%), nausea and vomiting (21%), constipation (21%), and mucositis (21%). In combination with 7 + 3: prolonged thrombocytopenia (19% vs 7%), prolonged neutropenia (3% vs 0%)^a^	During induction phase	Supportive care: Monitor blood counts frequently until recovery. Administer platelet transfusions as needed If platelet or neutrophil count does not recover to greater than or equal to 100 Gi/L and 0.5 Gi/L respectively within 14 days following the planned start date of the consolidation cycle, discontinue drug.
	Serious: 1) Hepatotoxicity including severe or fatal hepatic veno-occlusive disease (VOD) (5% ALFA trial GO arm—fatal in 50% of those afflicted) 2) Hemorrhage: grade 3–4 bleeding in 21% on ALFA trial GO arm, including fatal bleeding events (3%) (e.g., cerebral hematoma, intracranial hematoma, subdural hematoma) 3) Infusion related reactions (phase II studies reported one third of patients with a grade 3–4 infusion-related adverse event) [39]	1) Veno-occlusive disease occurred at a median time 9 days (range 2–298 days) 2) During the entire treatment period. 3) Infusion related reactions can occur during infusion and up to 24 h after, most commonly during the first infusion.	1) For total bilirubin > 2 × ULN or AST and/or ALT > 2.5 × ULN, hold drug until recovery of total bilirubin to ≤ 2 × ULN and AST and ALT ≤ 2.5 × ULN. For VOD, institute supportive care and discontinue drug. 2) Dose delay or permanent discontinuation. 3) Premedicate with a corticosteroid (e.g., 1 mg/kg methylprednisolone), acetaminophen 650 mg, and diphenhydramine (50 mg). Patients should be monitored until 1 h after infusion. If reaction occurs, interrupt infusion and treat with same dose of steroid, acetaminophen and/or antihistamine. Permanently discontinue treatment if severe or life-threatening reaction.
Gilteritinib [41]	Common (≥ 25%): transaminase increased (51%), myalgia/arthralgia (50%), fatigue/malaise (44%), fever (41%), mucositis (41%), edema (40%), rash (36%), non-infectious diarrhea (35%), dyspnea (35%), nausea (30%),, cough (28%), constipation (28%), and eye disorders (25%). Common grade 3–4 laboratory abnormalities ≥ 5%: hypophosphatemia (14%), increased ALT (13%), hyponatremia (12%), AST increased (10%), hypocalcemia (6%), increased CK (6%), and triglycerides increased (6%)	During the entire treatment period.	Assess blood counts and chemistries including creatinine phosphokinase at baseline, at least weekly for the first month, every other week for the second month, and once monthly for the duration of therapy. Any nonhematologic toxicity grade 3 of over, hold drug until toxicity resolves or improves to Grade 1 and resume at a dose of 80 mg.
	Serious: 1) Differentiation syndrome (3%) 2) QT prolongation > 500 ms (1%), increase from baseline QTc > 60 ms (7%) 3) Posterior Reversible Encephalopathy Syndrome (1%) 4) Pancreatitis (4%)	1) Differentiation syndrome was seen from 2 to 75 days after starting therapy. 2)–4) During the entire treatment period.	1) Steroids (Dexamethasone 10 mg BID) with taper and supportive care. Interrupt drug if severe signs/symptoms persist > 48 h. Resume when adverse events are ≤ grade 2. 2) Assess EKGs prior to initiation of treatment with gilteritinib, on days 8 and 15 of cycle 1, and prior to the start of the next two subsequent cycles. If QTc interval > 500 ms, interrupt drug and resume at 80 mg when QTc interval returns to within 30 ms of baseline or ≤ 480 ms. 3) Discontinue drug. 4) Hold drug until pancreatitis is resolved. Then resume at a dose of 80 mg.
Glasdegib [42]	Common adverse reactions and laboratory abnormalities (≥ 20% and ≥ 2% more common on glasdegib + LDAC arm): creatinine increased (96% vs. 80%), hyponatremia (54% vs. 41%), hypomagnesemia (33% vs. 23%), febrile neutropenia (31% vs. 22%), thrombocytopenia (30% vs. 27%), fatigue (36% vs. 32%), edema (30% vs. 20%), musculoskeletal pain (30% vs. 17%), nausea (29% vs. 12%), AST increased (28% vs. 23%), decreased appetite (21% vs. 7%), dysgeusia (21% vs. 2%), mucositis (21% vs. 12%), constipation (20% vs. 12%), and rash (20% vs. 7%).	Within the first 90 days of therapy. Muscle spasms and decreased appetite worsened after the first 90 days of therapy in some patients.	Monitor blood counts, electrolytes, renal, and hepatic function prior to initiation and at least once monthly for the first month. Monitor electrolytes and renal function once monthly for the duration of therapy. Check creatine kinase at baseline and as clinically indicated. Dose modifications: For grade 3 non-hematologic toxicity, hold glasdegib and/or LDAC until toxicity resolves or improves to Grade 1 and resume same dose of glasdegib or reduce to 50 mg. Discontinue glasdegib and LDAC for grade 4 nonhematologic toxicity.
	Serious: 1) QT prolongation > 500 ms (5%), increase from baseline QTc > 60 ms (4%) 2) Strongly embryotoxic, fetotoxic, and teratogenic	During the entire treatment period	1) Assess EKGs at baseline, after one week, and then once monthly for the next 2 months; repeat if abnormal. Avoid concomitant use with other QTc prolonging drugs. Avoid use of strong CYP3A4 inhibitors. If QTc interval > 500 ms, interrupt glasdegib and resume at 50 mg when QTc interval returns to within 30 ms of baseline or ≤ 480 ms. Permanently stop drug if there are signs or symptoms of life-threatening arrhythmia. 2) Must use contraception for females and males for at least 30 days after last dose. Pregnancy test must be done prior to initiating drug in women of reproductive potential.
Ivosidenib [61]	Common adverse reactions and laboratory abnormalities (≥ 25%)^d^: anemia (60%), hyponatremia (39%), fatigue (39%), hypomagnesemia (38%), leukocytosis (38%), arthralgia (36%), diarrhea (34%), dyspnea (33%), edema (32%), uric acid increased (32%), hypokalemia (31%), increased AST (27%), increased alkaline phosphatase (27%), nausea (31%), mucositis (28%), QT prolongation (26%), rash (26%), hypophosphatemia (25%)	During the entire treatment period.	Monitor blood counts and chemistries at baseline, at least weekly for the first month, once every other week for the second month, and once monthly for the duration of therapy. Monitor creatine phosphokinase weekly for the first month of therapy. Any non-hematologic toxicity grade ≥ 3, stop drug until resolves to grade 2 or lower. Resume drug at 250 mg once daily and can increase to 500 mg once daily if toxicities resolve to grade 1 or lower. If grade 3 or higher toxicity recurs, discontinue drug.
	Serious: 1) Differentiation syndrome (19% R/R patients; 13% grade ≥ 3, 25% newly-diagnosed patients; 11% grade ≥ 3) 2) QT prolongation > 500 msec (9%), increase from baseline QTc > 60 msec (14%) 3) Leukocytosis (8% grade ≥ 3 R/R patients, 7% grade ≥ 3 newly-diagnosed patients) 4) Guillain-Barré syndrome (< 1%)	1) Differentiation syndrome occurred as early as 1 day and up to 3 months after drug initiation. 2)-4) During the entire treatment period.	1) Steroids (Dexamethasone 10 mg BID) with taper and hemodynamic monitoring for at least 3 days. Interrupt drug if severe signs and/or symptoms persist > 48 h after steroid initiation. Resume when adverse events are ≤ grade 2. 2) Monitor ECGs at least once weekly for the first 3 weeks of therapy and then at least once monthly for the duration of therapy. If QTc interval > 500 ms, stop drug and resume at 250 mg when QTc interval returns to within 30 ms of baseline or ≤ 480 ms. Monitor EKG weekly for 2 weeks following resolution and consider re-escalating to 500 mg daily. Permanently stop drug if there are signs or symptoms of life-threatening arrhythmia. Avoid concomitant use with other QTc prolonging drugs. Avoid use of strong or moderate CYP3A4 inhibitors. Dose reduce ivosidenib to 250 mg daily if co-administration of a strong CYP3A4 inhibitor is unavoidable. 3) For WBC > 25 × 10^9^/L or absolute increase of > 15 × 10^9^/L from baseline, initiate treatment with hydroxyurea and/or leukapheresis and interrupt drug if leukocytosis does not improve. When leukocytosis resolves, resume ivosidenib. 4) Supportive care and discontinue drug permanently.
Midostaurin [28]	Common adverse events and laboratory abnormalities (≥ 25% and ≥ 2% more common on midostaurin arm): febrile neutropenia (83% vs. 81%), nausea (83% vs. 70%), ALT increased (71% vs. 69%), hypocalcemia (74% vs. 70%), mucositis (66% vs. 62%), vomiting (61% vs. 53%), headache (46% vs. 38%), petechiae (36% vs. 27%), musculoskeletal pain (33% vs. 31%), and epistaxis (28% vs. 24%).	Throughout the treatment period.	Supportive care: Monitor blood counts frequently and give antibiotics as clinically indicated until recovery. Any nonhematologic toxicity ≥ grade 3, interrupt Midostaurin until event has resolved to ≤ Grade 2, then resume at a dose of 50 mg twice daily. If tolerated, can increase to 100 mg twice daily.
	Serious: Pulmonary toxicity (interstitial lung disease or pneumonitis, with some reported fatal cases)	Throughout the treatment period.	Discontinue midostaurin in patients with signs or symptoms of interstitial lung disease or pneumonitis without an infectious etiology. Start steroids (Dexamethasone 10 mg BID) with taper, hemodynamic monitoring and supportive care until symptom resolution [43].
Venetoclax [44]	Common (≥ 30%)^e^: nausea, diarrhea, thrombocytopenia, constipation, neutropenia, febrile neutropenia, fatigue, vomiting, peripheral edema, pyrexia, pneumonia, dyspnea, hemorrhage, anemia, rash, abdominal pain, sepsis, back pain, myalgia, dizziness, cough, oropharyngeal pain, and hypotension. Common nonhematologic laboratory abnormalities (≥ 30%)^e^: hyperglycemia, hypocalcemia, hypoalbuminemia, hypokalemia, hyponatremia, hypophosphatemia, hyperbilirubinemia, hypomagnesemia, creatinine increased, bicarbonate decreased	Throughout the treatment period.	Supportive care: Monitor blood counts frequently and give antibiotics as clinically indicated until count recovery. If grade 4 neutropenia or thrombocytopenia: - Prior to remission: supportive care; transfuse blood products and administer prophylactic or treatment with antibiotics as indicated. - First occurrence after achieving remission and lasting at least 7 days: delay subsequent treatment cycle. Administer G-CSF if clinically indicated for neutropenia. Once toxicity grade 1 or 2, resume treatment at same dose in combination with HMA or LDAC. - Subsequent occurrences in cycles after remission and lasting 7 days or longer: delay subsequent treatment cycle. Administer G-CSF if clinically indicated for neutropenia. Once toxicity grade 1 or 2, resume treatment at same dose and reduce duration by 7 days for each subsequent cycle.
	Serious: 1) Tumor Lysis Syndrome 2) Neutropenia (96–100% experienced grade ≥ 3)	1) At initiation and during the ramp-up phase 2) Throughout the treatment period.	1) Prior to the first dose, premedicate with anti-hyperuricemic agents and ensure adequate hydration; continue during the ramp-up phase. All patients should have white blood cell count < 25 × 10^9^/L prior to initiation of drug. May have to cytoreduce prior to treatment. Monitor blood chemistries for TLS at pre-dose, 6 to 8 h after each new dose during ramp-up and 24 h after reaching final dose. Can consider increased laboratory monitoring and reduced starting dose for patients at higher risk of TLS. 2) See above.

^a^Definition of prolonged thrombocytopenia and neutropenia: platelets < 50 Gi/L or neutrophils < 0.5 Gi/L lasting past cycle day 42 in the absence of active leukemia

^b^Median time to platelet count ≥ 50 Gi/L and neutrophil count ≥ 0.5 Gi/L in patients with CR/CRi response after initial induction chemotherapy

^c^Adverse events as reported in relapsed and refractory MyloFrance 1 clinical trial

^d^Adverse events in patients with R/R AML

^e^Includes adverse reactions seen in combination with azacitidine or decitabine or LDAC. See prescribing information [44] for the number of adverse reactions for each combination individually

A phase I/II trial (NCT02287233) of venetoclax in combination with low-dose cytarabine (LDAC) in newly diagnosed patients led to a CR + CRi rate of 54% with a median duration of CR + CRi of 8.1 months (95% CI 5.3–14.9 months) and a median OS of 10.1 months (95% CI 5.7–14.2 months). In this trial, 89% of patients with a *NPM1* mutation achieved a CR or CRi. Patients with a *FLT3* mutation had a CR + CRi rate of 44% and those with TP53 mutations had CR + CRi rates of 30% [78, 87, 88].

For the LDAC combination, a dose of 600 mg venetoclax was well-tolerated following the 3-day ramp-up schedule. Adverse events in combination with LDAC were similar to those previously listed for the venetoclax and HMA trial. Additional grade ≥ 3 adverse drug reactions or laboratory abnormalities in > 5% of patients included hypokalemia (20%), hypocalcemia (16%), hemorrhage (15%), and hyponatremia (11%). The incidence of TLS was 3%.

Both options of venetoclax plus a HMA or LDAC are available for patients greater than 75 years or with comorbidities that preclude use of intensive chemotherapy. The confirmatory phase III trials comparing venetoclax and azacitidine to azacitidine alone (VIALE-A) (NCT02993523) and venetoclax and LDAC to LDAC alone (VIALE-C) (NCT03069352) are ongoing to confirm the clinical benefit of the combination therapies. Based on the preliminary response rate and survival data, the HMA backbone is preferred unless the patient has previously received a HMA for MDS. There are no clear data to support the superiority of one HMA over another, although there is more data with the azacitidine combination and this was the regimen chosen for the phase III trial.

### Glasdegib (Daurismo) [42, 85] combination with low-dose cytarabine: newly diagnosed AML ≥ 75 years or comorbidities

Activation of the Hedgehog (Hh) signaling pathway leads to the release of proteins that translocate to the nucleus and promote transcription of selected target genes. Aberrant activation of Hh and its downstream intermediaries occurs at the level of the cancer stem cell and may confer drug resistance by maintaining stem cell quiescence and survival. Preclinical studies targeting Hh downstream proteins such as smoothened (SMO) or glioma-associated protein (GLI) with small molecule inhibitors demonstrate that Hh inhibition decreases the presence of leukemic stem cells [74, 76].

A randomized phase II clinical study, BRIGHT AML 1003, studied glasdegib in combination with LDAC compared to LDAC alone. Similar to the venetoclax combinations, glasdegib in combination with LDAC showed clinical activity in AML patients who were older or had comorbidities prohibiting tolerability of intensive treatment, with CR + CRi rates in the combination group of 25% versus 5% in the LDAC alone group [89]. The median OS was 8.3 months with glasdegib + LDAC compared to 4.3 months with LDAC alone (HR, 0.46, *p* = 0.0002). In an exploratory subgroup analysis, the authors found an enhanced effect on OS in patients with good to intermediate risk AML [75].

The most common (≥ 20%) adverse reactions and laboratory abnormalities ≥ 2% higher on the glasdegib + LDAC arm compared to the LDAC alone arm are listed in Table 2 [42].

Typically, the choice of LDAC is reserved for frail unfit patients who prefer treatment over best supportive care. Treatment with LDAC and either venetoclax or glasdegib is approved for patients 75 years and older or with significant comorbidities preventing use of more toxic therapy. Although the overall response rates favor LDAC/venetoclax, the CR rates with both regimens are similar in this patient population, and there has been no head to head comparison between the two regimens. The ongoing phase III trial, BRIGHT AML10109 (NCT 03416179), will determine whether glasdegib therapy may have a broader impact, as it investigates both intensive chemotherapy with 7 + 3 with or without glasdegib and non-intensive therapy with azacitidine with or without glasdegib in patients with newly diagnosed AML.

### CPX-351 (Vyxeos) [34, 90]: newly diagnosed AML-MRC and t-AML

The World Health Organization (WHO) classification of myeloid neoplasm and acute leukemia was updated in 2008 to include AML with myelodysplasia-related changes (AML-MRC) and therapy-related myeloid neoplasms (t-MNs) [91]. In 2016, both names were retained; however, subtleties were added to reflect a more accurate prognostic significance. For AML-MRC, patients must still have a history of myelodysplastic syndrome (MDS) or MDS/MPN and have evolved to AML, have a category-defining cytogenetic abnormality, or have dysplasia in 50% of the cells in two or more lineages. Currently, multi-lineage dysplasia alone is not enough to meet criteria for this category when an NPM1 mutation or bilallelic CEBPA are present. Deletion 9(q) has also been removed as a category-defining cytogenetic abnormality. For t-MNs, patients may either have t-MDS or t-AML [92]. With the update to the classification systems comes a greater recognition of identification for prognostic purposes and has implications for treatment choices.

Patients with t-AML or AML-MRC tend to be older and have more comorbidities. As many of these patients have received previous cytotoxic therapies, they may have pre-existing depletion of hematopoietic reserves which, in turn, may be associated with decreased CR rates and inferior OS compared with de novo AML. Patients with AML-MRC that is non-MDS mediated have worse outcomes, independent of age and cytogenetics, but at least in part related to molecular mutations in diverse tumor suppressor genes such as TP53 [93].

CPX-351 has demonstrated efficacy in patients with treatment-related or secondary AML. It is a dual-drug liposomal encapsulation of daunorubicin and cytarabine and has a fixed 1:5 M ratio of these drugs. Each unit of CPX-351 contains 0.44 mg daunorubicin and 1 mg cytarabine and the liposomal membrane is a 7:2:1 ratio of distearylphosphatidylcholine, distearylphosphatidylgycerol, and cholesterol. This formulation enables intracellular delivery of the synergistic drug ratio, which improves the uptake into leukemic cells relative to normal cells. Compared with conventional 7 + 3, the ratio of the two drugs is maintained for more than 24 h in plasma and bone marrow [73]. In this regard, CPX-351 overcomes several resistance mechanisms by entering cells as liposomes, thereby bypassing drug efflux pumps, and providing prolonged intracellular exposure [94]. First-in-human studies in patients with acute lymphoblastic leukemia (ALL) and AML detected a median half-life of 21.9 h for the daunorubicin and 31.1 h for the cytarabine components. Pharmacokinetic data revealed that both drugs and their metabolites were present systemically more than 7 days after the last dose, supporting the notion that the liposomal formulation may confer an extended duration of exposure to relatively high levels of both drugs. Toxicities were consistent with those of standard 7 + 3; however, frequency of events increased with higher doses and dose-limiting toxicities included prolonged cytopenias, hypertensive crisis, and congestive heart failure [94].

Phase II studies of CPX-351 produced higher overall response rates compared to standard 7 + 3 (66.7% vs 51.2%), but the differences in EFS and OS were not statistically significant. However, there was improved OS and EFS in the subgroup of patients aged 60–75 with secondary AML when compared with 7 + 3 [95]. These results led to a phase III clinical trial of CPX-351 compared with standard 7 + 3 in previously untreated AML patients 60–75 years of age with t-AML, AML with antecedent MDS, chronic myelomonocytic leukemia (CMML), or de novo AML with WHO-defined MDS-related cytogenetic abnormalities. CPX-351 led to significant improvements in remission rates, EFS, and OS when compared with 7 + 3. The combined CR + CRi rates in the CPX-351 patients with one induction cycle were 55.2% versus 34.0% in the 7 + 3 group. For those who required a second induction cycle, the CR + CRi rates were again higher on the CPX-351 arm compared to the 7 + 3 arm (47.7% vs. 33.3%) [71]. While there was a higher remission rate in the CPX-351 arm, the median duration of remission was similar for both arms. The proportion of patients who proceeded to allo-HSCT was 34% vs. 25% with CPX-351 and 7 + 3, respectively. An exploratory landmark analysis looking at survival from the time of allo-HSCT favored CPX-351 (HR 0.46 [95% CI 0.24–0.89], *p* = 0.009). A subgroup analysis showed that patients with prior HMA exposure did not seem to benefit, while those who had not received an HMA prior derived an OS benefit. Although the numbers were small, the 11 patients with previous CMML appeared to benefit from the therapy [71].

Early mortality rates at 30 and 60 days were not significantly different between the two arms, although there was a trend toward decreased mortality in the CPX-351 arm compared with 7 + 3 (5.9% vs. 10.6% at 30 days and 13.7% vs. 21.2% at 60 days, respectively) [71]. Important toxicities with CPX-351 included a longer time to neutrophil (35 vs. 29 days) and platelet (36.5 vs. 29 days) recovery, with an increased number of bleeding events in the CPX-351 cohort vs. 7 + 3 (all-grade 74.5% vs. 59.6%, grade ≥ 3 11.8% vs. 8.6%), related at least in part to persistence of CPX-351 liposomes in the plasma with resultant prolonged drug exposure [71]. Grade ≥ 3 adverse reactions during induction in > 10% of patients were similar in both groups (listed in Table 2); however, the incidences of pneumonia, fungal infection, and upper respiratory tract infections were slightly higher in the CPX-351 arm [34]. There was also a greater number of grade 5 infections in the CPX-351 arm compared to the 7 + 3 arm (7% vs. 3%) [71].

Based on the survival benefit demonstrated with CPX-351 in patients with t-AML and AML-MRC, this approach can be considered in newly diagnosed patients who are able to tolerate intensive induction chemotherapy. While the drug is approved for all adult patients, the age range in the study was only 60–75 years. FDA extrapolated the efficacy results to younger adult patients based on expectation that the biology of t-AML and AML-MRC are consistent across the adult patient population; furthermore, safety results in younger patients did not show any concerns [90]. A phase III clinical trial plans to determine the benefit of CPX-351 over 7 + 3 in newly diagnosed AML patients 18 years and older with intermediate or adverse-risk genetics (NCT03897127).

### Gemtuzumab ozogamicin (Mylotarg) [38]: newly diagnosed and relapsed/refractory CD33-positive AML

The majority of AML cells express varying amounts of the CD33 surface antigen (estimated > 80% of patients with AML) [96]. Gemtuzumab ozogamicin (GO) is a humanized anti-CD33 monoclonal antibody linked to the cytotoxic agent *N*-acetyl calicheamicin [12]. Preliminary research found early internalization after antigen binding followed by intracellular release led to the delivery of the therapeutic agent in CD33-expressing leukemic cells [96, 97]. GO initially received accelerated approval by the FDA in 2000 on the basis of a CR + CRp rate of 30% (CR rate 16%) across three open-label phase II trials in patients with first relapse of CD33^+^ AML [39]. These initial studies dosed GO at 9 mg/m^2^ 14 days apart for up to three doses. Several post-marketing reports revealed cases of fatal anaphylaxis, adult respiratory distress syndrome, and hepatotoxicity, especially venoocclusive disease (VOD) in patients treated with GO, leading to labeling revisions and initiation of a registration surveillance program.

To confirm clinical benefit, the Southwest Oncology Group (SWOG) conducted Study S0106, a phase III trial comparing 7 + 3 induction with or without one dose of GO at 6 mg/m^2^ on day 4. Unfortunately, the addition of GO to induction or post-consolidation therapy failed to show an improvement in CR rate, relapse-free survival (RFS), or OS. In addition, the number of induction deaths was higher in the GO group [98]. As such, GO was voluntarily withdrawn from the United States (U.S.) market in 2010 [12].

Of note, researchers hypothesized that repeated lower doses of GO may be able to increase the internalization process of the drug into leukemia cells while enhancing safety [82]. This is referred to as the “fractionated” dose and schedule of GO given that it consists of one 9 mg/m^2^ dose divided into three separated dose fractions of 3 mg/m^2^ on days 1, 4, and 7. Of note, exposure-response relationships in the three single-arm trials of GO 9 mg/m^2^ 14 days apart showed that increased Cmax was significantly correlated with a higher risk of VOD, but not higher CR rates. A meta-analysis of GO monotherapy conducted by FDA across multiple phase I and II studies showed that CR rate was more favorable, there were no cases of VOD, and early mortality was lower using the 3 mg/m^2^ fractionated dose and schedule when compared to both the 9 mg/m^2^ and 6 mg/m^2^ unfractionated regimens [40].

When tested in the first relapse setting in adults with CD33-positive de novo AML in Study MyloFrance 1, the fractionated dose-schedule of GO 3 mg/m^2^ days 1, 4, and 7 as monotherapy showed a 26% CR and 33% CR + CRp rate [82]. While there were no differences in CR rates based on age or cytogenetic risk, blast clearance by day 15 (less than 5% blasts in the bone marrow) was associated with better rates of CR/CRp. This study also explored the expression of the multidrug resistance family of ABC proteins on viable cells as a potential predictive determinant of clinical drug resistance. Expression of ABCB1 (P-glycoprotein or Pgp) and/or ABCC1 (multidrug resistance protein 1 or MRP1) activities were strongly associated with a poor clinical response and treatment failure; however, the relationship between multidrug resistance protein activity and clinical outcome with GO will require assessment in larger studies to determine the utility of these proteins as biomarkers for treatment success.

Common adverse reactions on study MyloFrance 1 are displayed in Table 2. No episodes of VOD occurred, but only seven patients proceeded to HSCT after treatment with GO (three allogeneic, four autologous) [82].

The results of MyloFrance 1 formed the basis of FDA’s re-approval of GO for CD33-positive R/R AML [40]. Treatment of R/R CD33-expressing AML with GO as a single agent is a treatment option but given the lack of randomized data in this setting, it is not known whether GO provides more beneficial outcomes when compared to other available salvage therapies. Furthermore, the treating physician must take into consideration the risk of VOD in patients designated for allogeneic HSCT. Although VOD was not observed in Study MyloFrance 1, the number of patients who went to HSCT was small. Furthermore, the protocol recommended a minimum delay of 90 days between GO therapy and HSCT. Of 19 patients with CR + CRp responses, 18 received post-remission therapy with HiDAC and the mean time between GO infusion and HSCT was 5 months (range 3.7–7.2 months) [82].

In the upfront setting, the phase III study ALFA-0701 was conducted across France randomizing patients 50–70 years of age with de novo AML to standard induction chemotherapy with or without GO at 3 mg/m^2^ on days 1, 4, and 7 of induction cycle 1 and then day 1 of two consolidation cycles. This trial ultimately supported the return of GO to the United States market [12]. Published results showed that CR and CRp was 81% in the GO group versus 75% in the control arm and that EFS was significantly prolonged on the GO arm (HR 0.58, 0.43–0.78; *p* = 0.0003) [77]. Benefits were more apparent in patients with favorable and intermediate risk cytogenetics, and those positive for the *FLT3*-ITD mutation, while patients with adverse risk cytogenetics did not appear to benefit (HR 1.03 [95% CI 0.50–2.13]). The number of induction deaths was similar between the groups. The GO group was associated with persistent thrombocytopenia after chemotherapy (19% vs. 7%) and more hepatotoxicity, with VOD incidence of 2% during induction and 5% overall (including three fatal cases) during or following treatment, including later allogeneic HSCT (see Table 2) [12, 38]. Thus, GO carries a boxed warning for hepatotoxicity, including severe or fatal hepatic VOD.

A meta-analysis of five randomized controlled trials adding GO to induction chemotherapy (*n* = 3325) [77, 98–101], including ALFA-0701 and S0106, demonstrated improved RFS (HR = 0.84 [95% CI 0.76–0.92]; *p* = 0.0003) and marginally improved OS (HR = 0.90 [95% CI 0.82–0.98]; *p* = 0.01) in patients receiving GO. Enhanced benefit was again observed in patients with favorable and intermediate risk cytogenetics, with an apparent lack of benefit in patients with adverse risk cytogenetics (odds ratio 1.03 [95% CI 0.85–1.24]) [102]. Although there was no difference in 30-day induction mortality with a single dose of GO at 3 mg/m^2^ versus three fractionated doses of GO in ALFA-0701, there was a trend toward decreased induction mortality with the former. Both doses, however, were favorable when compared to the 6 mg/m^2^ dose of GO.

GO can be considered a therapeutic option for newly diagnosed CD33^+^ AML patients treated with standard cytotoxic therapy, particularly in those with favorable or intermediate risk cytogenetics. Given that the benefit of GO was not apparent in patients with adverse risk cytogenetics across multiple trials, it is not recommended for use in these patients. Furthermore, given the preference for allogeneic HSCT in CR1 for patients with intermediate risk cytogenetics, some may advise against the use of GO even in intermediate risk patients. Of note, only 13% of patients assigned to GO on the ALFA-0701 trial underwent HSCT in first CR/CRp [103], so it is unclear whether a higher incidence of VOD would have been observed had more patients underwent transplantation.

The phase III EORTC-GIMEMA AML-19 trial compared GO against best supportive care in older newly diagnosed AML patients (> 75 or 61–75 years with poor performance scores or unwilling to receive standard chemotherapy). This trial used a distinct dose and schedule of GO of 6 mg/m^2^ on day 1 and 3 mg/m^2^ on day 8, with up to eight courses of 2 mg/m^2^ on day 1 every 4 weeks. There was a response and survival benefit for patients receiving GO with an overall CR + CRi rate of 27% (CR rate 15%) and median OS of 4.9 months, compared with 3.6  months with best supportive care (hazard ratio 0.69; 95% CI, 0.53 to 0.90; *p* = 0.005). Subgroup analyses demonstrated a greater OS benefit in patients with higher CD33 expression, female sex, and like prior studies, favorable/intermediate-risk cytogenetics. In this trial, toxicities were comparable between the arms, with no cases of VOD on the GO arm [83].

Monotherapy with GO could be considered in older adults with newly diagnosed CD33^+^ AML based on the results of AML-19. However, it is unclear how well GO monotherapy would perform against other commonly used standard of care regimens for this patient population, such as HMA and LDAC-based regimens.

## Conclusion

The last 2 years have been a very active period for the clinical testing and FDA approval of diverse molecularly targeted treatments in AML, with several new agents and additional clinical trials currently underway. As a result of these drug developments, more options are now available for patients with various subtypes of AML, and in particular, older patients or those with comorbidities. Some of these new drugs are more promising than others with respect to response rate and safety profile, and a practical conversation with patients must occur regarding balancing efficacy and toxicity to maximize quality and quantity of life.

At the present time, there are not enough data to know how best to use these newly approved drugs in a particular sequence or combination. The full application of these agents to AML patients with and without evidence of the specific molecular targets for which the drugs have been developed will require randomized clinical trials that compare these agents with currently accepted approaches. Combinations of targeted agents with HMAs and standard cytotoxic therapies are currently under investigation in diverse stages of disease, including post-chemotherapy and/or post-transplant maintenance, and will shed light on how to sequence these agents to maximize OS, EFS, and quality of life. Combinations of these new agents with mechanistically distinct agents that are not yet approved for AML, such as other targeted small molecule inhibitors and immunotherapies, are future considerations that need to be investigated through scientifically rigorous clinical-correlative trials. Finally, with continued bidirectional investigations, we need to learn how AML cells develop resistance to each of these new agents (e.g., isotype switching in response to IDH-1 or -2 inhibitors, rebound MCL-1 expression induced by venetoclax) and how to abrogate or overcome such resistance through rational combinations and sequences.

## Data Availability

The material supporting the information of this review has been included within the article.

## Abbreviations

7 + 3: 7 days of cytarabine and 3 days of an anthracycline
ABCB1: ATP binding cassette subfamily B member 1
ABCC1: ATP binding cassette subfamily C member 1
ALL: Acute lymphoblastic leukemia
Allo-HSCT: Allogenic hematopoietic transplant
AML: Acute myeloid leukemia
AML-MRC: Acute myeloid leukemia with myelodysplasia-related changes
BCL-2: B cell lymphoma 2
CEBPA: CCAAT enhancer binding protein alpha
CI: Confidence interval
CLL: Chronic lymphocytic leukemia
CMML: Chronic myelomonocytic leukemia
CR: Complete remission
CRh: CR with partial hematologic recovery
CRi: CR with incomplete count recovery
CRp: CR with incomplete platelet counts
DFS: Disease-free survival
DS: Differentiation syndrome
EFS: Event free survival
FDA: Food and Drug Administration
FLAG-IDA: Fludarabine, cytarabine, idarubicin and granulocyte-colony stimulating factor
FLT3: Fms related tyrosine kinase 3
GLI: Glioma-associated protein
GO: Gemtuzumab ozogamicin
Hh: Hedgehog
HMA: Hypomethylating agent
HR: Hazard ratio
IDH: Isocitrate dehydrogenase
ITD: Internal tandem duplication mutation
KIT: KIT proto-oncogene receptor tyrosine kinase
LDAC: Low-dose cytarabine
MCL-1: Myeloid cell leukemia-1
MDS: Myelodysplastic syndrome
MEC: Mitoxantrone, etoposide and cytarabine
MPN: Myeloproliferative neoplasms
MRD: Minimal residual disease
NPM1: Nucleophosmin 1
NR: Not reached
OS: Overall survival
PDGF-Rβ: Platelet derived growth factor receptor-beta
PKC: Protein kinase C
QTc: Correct QT
R/R: Relapsed or refractory
SMO: Smoothened protein
SWOG: Southwest Oncology Group
t-AML: Therapy-related acute myeloid leukemia
TKD: Tyrosine kinase domain
TLS: Tumor lysis syndrome
t-MN: Therapy-related myeloid neoplasms
TP53: Tumor protein p53
U.S.: United States
UGT1A1: Uridine diphosphate glucuronosyltransferase glucuronosyltransferase 1 family, polypeptide A1
USPI: United States package insert
VEGFR-2: Vascular endothelial growth factor receptor 2
VOD: Venoocclusive disease
WHO: world health organization
